# Supplementary material for: Natural History of Oral HPV Infection among Indigenous South Australians
Source: Viruses. 2023 Jul 18;15(7):1573. doi: 10.3390/v15071573 (PMC10385757; doi:10.3390/v15071573)
Supplement: Supplementary file 1 [file viruses-15-01573-s001.zip › viruses-2452862-supplementary.pdf]

Table S1: Baseline sample characteristics by valid and invalid saliva sample

	Valid [a] n=473	Invalid [b] n=536
**Age group (years)**		
≥ 37	56.8 (52.3-61.3)	48.1 (44.2-52.7)
< 37	43.2 (38.7-47.7)	51.6 (47.3-55.8)
**Sex**		
Male	**26.3 (22.3-30.2)**	**39.9 (35.7-44.0)**
Female	**73.7 (69.8-77.7)**	**60.1 (56.0-64.3)**
**Geographic location**		
Non-metropolitan	60.0 (55.5-64.4)	65.0 (61.0-69.1)
Metropolitan	40.0 (35.6-44.5)	35.0 (30.9-39.0)
**Education level**		
High school or less	63.8 (59.4-68.2)	72.1 (68.1-75.9)
Trade or over	36.2 (31.8-40.6)	27.9 (24.1-31.9)
**Income**		
Centrelink	72.7 (68.6-76.7)	79.0 (75.5-82.5)
Job	27.3 (23.3-31.4)	21.0 (17.5-24.5)
**Health care card**		
Yes	76.5 (72.5-80.4)	81.0 (77.6-84.4)
No	23.5 (19.6-27.4)	19.0 (15.6-22.4)
**Car ownership**		
No	40.3 (35.8-44.7)	48.3 (44.1-52.6)
Yes	59.7 (55.3-64.2)	51.7 (47.4-55.9)
**Smoke status**		
Current smoker	57.8 (53.2-62.4)	61.1 (56.8-65.3)
Ex-smoker	14.3 (11.0-17.5)	9.7 (7.1-12.3)
Never smoker	27.9 (23.7-32.1)	29.2 (25.3-33.2)
**Recreational drug use**		
Current user	17.2 (13.8-20.6)	24.0 (20.3-27.6)
Ex-user	30.8 (26.6-35.0)	35.8 (31.8-39.9)
Never use	**52.0 (47.5-56.6)**	**40.2 (36.0-44.4)**
**Had oral sex**		
Yes	75.5 (68.5-79.6)	64.1 (59.8-68.3)
No	24.5 (20.4-31.2)	35.9 (30.7-40.2)
**Self-rated general health**		
Fair/Poor	75.7 (71.8-79.6)	79.4 (75.9-82.8)
Excel/very good/Good	24.3 (20.4-28.2)	20.6 (17.2-24.1)
**Self-rated oral health**		
Fair/Poor	61.6 (57.2-66.9)	70.7 (66.8-74.6)
Excel/very good/Good	38.4 (33.9-42.8)	29.3 (25.4-33.2)

Note: a: β-globin positive at 3 timepoints. b: including lost to follow-up at 12- and/or 24-month or β-globin negative at one of three timepoints. Bold were donated as statistically significant differences.

Table S2. Persistence of the same HPV type infection at baseline, 12- and 24-month follow-up among Indigenous Australian adults (n=45).

**HPV type**				**Number**	**% (95% CI)**
	**Baseline**	**12 months**	**24 months**		
13	√	√	√	15	33.3 (19.0-47.7)
16	√	√	√	4	8.9 (0.2-17.5)
18	√	√	√	1	2.2 (0.0-6.7)
32	√	√	√	16	35.6 (21.0-50.1)
35	√	√	√	1	2.2 (0.0-6.7)
45	√	√	√	1	2.2 (0.0-6.7)
51	√	√	√	1	2.2 (0.0-6.7)
53	√	√	√	1	2.2 (0.0-6.7)
62	√	√	√	1	2.2 (0.0-6.7)
66	√	√	√	2	4.4 (0.0-10.7)
72	√	√	√	1	2.2 (0.0-6.7)
73	√	√	√	1	2.2 (0.0-6.7)

Notes: '√' HPV test positive.

Table S3. Persistence of the different HPV type infection at baseline, 12- and 24-month follow-up among Indigenous Australian adults (n=11).

**HPV type**			**Number**	**% (95% CI)**
**Baseline**	**12 months**	**24 months**		
**69**	**32**	**69**	1	9.1 (1.6-37.7)
**32**	**32**	**26**	1	9.1 (1.6-37.7)
**32**	**32**	**42**	2	18.2 (5.1-47.7)
**84**	**32**	**84**	1	9.1 (1.6-37.7)
**32**	**32**	**69**	1	9.1 (1.6-37.7)
**32**	**51**	**82**	1	9.1 (1.6-37.7)
**59**	**66**	**32**	1	9.1 (1.6-37.7)
**6**	**13**	**13**	1	9.1 (1.6-37.7)
**72**	**72**	**62**	1	9.1 (1.6-37.7)
**39**	**53**	**53**	1	9.1 (1.6-37.7)

Table S4. Multivariable association between oral HPV 13/32 infection prevalence and risk factors among Indigenous Australian adults (n=993).

	Model 1	Model 2	Model 3	Model 4	Model5
	Prevalence ratio (95% CI)				
Age group (years)					
≥ 37	0.97 (0.82-1.15)	0.97 (0.83-1.15)	0.90 (0.75-1.08)	0.95 (0.79-1.15)	0.90 (0.75-1.09)
< 37	ref	ref	ref	ref	ref
Sex					
Male	0.93 (0.79-1.11)	0.94 (0.79-1.12)	0.93 (0.76-1.14)	0.92 (0.75-1.14)	0.89 (0.73-1.10)
Female	ref	ref	ref	ref	ref
Geographic location					
Non-metropolitan	**1.73 (1.42-2.10)**	**1.74 (1.42-2.13)**	**1.81 (1.44-2.28)**	**1.79 (1.41-2.26)**	**1.81 (1.43-2.28)**
Metropolitan	ref	ref	ref	ref	ref
Education level					
High school or less	1.09 (0.91-1.30)	1.01 (0.84-1.21)	0.91 (0.75-1.12)	0.93 (0.76-1.14)	0.93 (0.76-1.14)
Trade or over	ref	ref	ref	ref	ref
Income					
Centrelink	1.04 (0.86-1.26)	0.99 (0.77-1.29)	1.04 (0.78-1.38)	1.01 (0.75-1.35)	1.02 (0.75-1.39)
Job	ref	ref	ref	ref	ref
Health care card					
Yes	0.96 (0.79-1.17)	0.89 (0.70-1.13)	0.84 (0.66-1.08)	0.88 (0.68-1.13)	0.85 (0.65-1.10)
No	ref	ref	ref	ref	ref
Car ownership					
No	**1.27 (1.08-1.49)**	1.20 (0.99-1.43)	1.11 (0.90-1.36)	1.13 (0.91-1.39)	1.12 (0.91-1.38)
Yes	ref	ref	ref	ref	ref
Smoke status					
Current smoker	0.86 (0.72-1.04)		0.86 (0.70-1.07)	0.87 (0.70-1.08)	0.87 (0.70-1.08)
Ex-smoker	1.01 (0.78-1.32)		1.06 (0.79-1.41)	1.03 (0.77-1.38)	1.03 (0.77-1.37)
Never smoker	ref		ref	ref	ref
Recreational drug use					
Current user	0.97 (0.79-1.19)		0.99 (0.77-1.28)	1.01 (0.78-1.31)	1.03 (0.80-1.33)
Ex-user	0.83 (0.68-1.00)		0.86 (0.68-1.09)	0.87 (0.69-1.11)	0.88 (0.70-1.11)
Never use	ref		ref	ref	ref
Had oral sex					
Yes	**0.77 (0.64-0.91)**		0.86 (0.71-1.06)	0.86 (0.70-1.05)	0.87 (0.71-1.07)
No	ref		ref	ref	ref
Self-rated general health					
Excel/very good/Good	**1.46 (1.15-1.84)**			1.31 (0.98-1.74)	1.31 (0.98-1.74)
Fair/Poor	ref			ref	ref
Self-rated oral health					
Excel/very good/Good	1.08 (0.90-1.29)			0.93 (0.75-1.14)	0.94 (0.76-1.15)
Fair/Poor	ref			ref	ref
Ever received HPV vaccination					
No	1.18 (0.86-1.62)				1.43 (0.99-2.05)
Don't know	0.98 (0.70-1.38)				1.32 (0.91-1.93)
Yes	ref				ref

Notes: **Model 1**: crude model; **Model 2**: adjusted for the sociodemographic factors (age, gender, geographic location, education level, income, health care card holder, car ownership); **Model 3**: plus adjusting for health-related behaviours (smokes and recreational drug status, and sexual behaviours); **Model 4**: plus adjusting for self-rated general and oral health; **Model 5** (full model): plus adjusting for self-reported HPV vaccination status. Bold were donated as statistically significant differences.

Table S5. Multivariable association between oral HPV 16/18 infection prevalence and risk factors among Indigenous Australian adults (n=993).

	Model 1	Model 2	Model 3	Model 4	Model5
	Prevalence ratio (95% CI)				
Age group (years)					
≥ 37	1.05 (0.59-1.86)	0.93 (0.52-1.68)	0.94 (0.50-1.77)	1.02 (0.53-1.96)	1.02 (0.53-1.95)
< 37	ref	ref	ref	ref	ref
Sex					
Male	0.99 (0.54-1.81)	1.00 (0.53-1.88)	0.76 (0.38-1.53)	0.73 (0.37-1.48)	0.73 (0.36-1.47)
Female	ref	ref	ref	ref	ref
Geographic location					
Non-metropolitan	0.33 (0.18-0.60)	**0.36 (0.19-0.66)**	**0.30 (0.16-0.58)**	**0.29 (0.15-0.56)**	**0.29 (0.14-0.56)**
Metropolitan	ref	ref	ref	ref	ref
Education level					
High school or less	0.64 (0.36-1.14)	0.71 (0.38-1.32)	0.75 (0.38-1.46)	0.76 (0.39-1.49)	0.76 (0.39-1.49)
Trade or over	ref	ref	ref	ref	ref
Income					
Centrelink	1.11 (0.56-2.21)	1.05 (0.42-2.58)	0.77 (0.31-3.73)	1.76 (0.30-1.92)	0.76 (0.30-1.92)
Job	ref	ref	ref	ref	ref
Health care card					
Yes	1.19 (0.56-2.52)	1.49 (0.59-3.78)	1.45 (0.57-3.73)	1.47 (0.58-3.75)	1.47 (0.58-3.74)
No	ref	ref	ref	ref	ref
Car ownership					
No	0.75 (0.42-1.35)	0.88 (0.46-1.66)	1.02 (0.51-2.03)	1.05 (0.53-2.09)	1.05 (0.53-2.09)
Yes	ref	ref	ref	ref	ref
Smoke status					
Current smoker	0.71 (0.35-1.41)		-	-	-
Ex-smoker	1.28 (0.52-3.13)		-	-	-
Never smoker	ref		-	-	-
Recreational drug use					
Current user	0.69 (0.28-1.69)		1.00 (0.38-2.66)	1.02 (0.38-2.71)	1.02 (0.38-2.71)
Ex-user	1.43 (0.77-2.63)		1.93 (0.95-3.23)	1.96 (0.96-3.98)	1.95 (0.96-3.97)
Never use	ref		ref	ref	ref
Had oral sex					
Yes	1.02 (0.52-1.97)		0.59 (0.28-1.23)	0.58 (0.28-1.22)	0.58 (0.27-1.22)
No	ref		ref	ref	ref
Self-rated general health					
Excel/very good/Good	1.13 (0.55-2.30)			1.39 (0.55-3.48)	1.39 (0.55-3.48)
Fair/Poor	ref			ref	ref
Self-rated oral health					
Excel/very good/Good	1.12 (0.60-2.07)			1.12 (0.52-2.42)	1.12 (0.52-2.42)
Fair/Poor	ref			ref	ref
Ever received HPV vaccination					
No	0.67 (0.26-1.71)				-
Don't know	0.83 (0.32-2.18)				-
Yes	ref				-

Notes: **Model 1**: crude model; **Model 2**: adjusted for the sociodemographic factors (age, gender, geographic location, education level, income, health care card holder, car ownership); **Model 3**: plus adjusting for health-related behaviours (smokes and recreational drug status, and sexual behaviours); **Model 4**: plus adjusting for self-rated general and oral health; **Model 5** (full model): plus adjusting for self-reported HPV vaccination status. Bold were donated as statistically significant differences.

Table S6. Multivariable association between oral hr-HPV infection prevalence and risk factors among Indigenous Australian adults (n=993).

	Model 1	Model 2	Model 3	Model 4	Model5
	Prevalence ratio (95% CI)				
Age group (years)					
≥ 37	0.97 (0.68-1.39)	0.91 (0.63-1.30)	1.01 (0.69-1.50)	1.12 (0.76-1.65)	1.12 (0.76-1.65)
< 37	ref	ref	ref	ref	ref
Sex					
Male	1.14 (0.78-1.64)	1.10 (0.76-1.60)	1.07 (0.71-1.61)	0.96 (0.64-1.43)	0.96 (0.64-1.43)
Female	ref	ref	ref	ref	ref
Geographic location					
Non-metropolitan	**0.28 (0.20-0.42)**	**0.29 (0.19-0.42)**	**0.28 (0.18-0.43)**	**0.29 (0.19-0.44)**	**0.29 (0.19-0.44)**
Metropolitan	ref	ref	ref	ref	ref
Education level					
High school or less	0.82 (0.55-1.16)	0.96 (0.65-1.42)	0.87 (0.58-1.31)	0.96 (0.64-1.145)	0.96 (0.64-1.145)
Trade or over	ref	ref	ref	ref	ref
Income					
Centrelink	1.04 (0.68-1.59)	0.92 (0.54-1.56)	0.89 (0.50-1.57)	0.85 (0.49-1.49)	0.85 (0.49-1.49)
Job	ref	ref	ref	ref	ref
Health care card					
Yes	1.23 (0.77-1.97)	1.60 (0.90-2.83)	1.77 (0.96-3.25)	1.63 (0.90-2.95)	1.63 (0.90-2.95)
No	ref	ref	ref	ref	ref
Car ownership					
No	0.71 (0.49-1.03)	0.74 (0.50-1.10)	0.77 (0.49-1.20)	0.83 (0.55-1.27)	0.83 (0.55-1.27)
Yes	ref	ref	ref	ref	ref
Smoke status					
Current smoker	0.92 (0.59-1.41)		0.73 (0.44-1.20	-	-
Ex-smoker	1.62 (0.94-2.77)		1.31 (0.74-2.32)	-	-
Never smoker	ref		ref	-	-
Recreational drug use					
Current user	1.45 (0.89-2.36)		1.69 (0.96-3.00)	1.55 (0.91-2.66)	1.55 (0.91-2.66)
Ex-user	**1.77 (1.18-2.67)**		**2.01 (1.23-3.28)**	**1.75 (1.22-3.04)**	**1.75 (1.22-3.04)**
Never use	ref		ref	ref	ref
Had oral sex					
Yes	**1.58 (1.48-2.28)**		**1.10 (1.10-1.91)**	**1.16 (1.06-1.63)**	**1.16 (1.06-1.63)**
No	ref		ref	ref	ref
Self-rated general health					
Excel/very good/Good	0.83 (0.55-1.24)			0.98 (0.59-1.63)	0.98 (0.59-1.63)
Fair/Poor	ref			ref	ref
Self-rated oral health					
Excel/very good/Good	1.02 (0.70-1.50)			1.16 (0.73-1.83)	1.16 (0.73-1.83)
Fair/Poor	ref			ref	ref
Ever received HPV vaccination					
No	0.80 (0.52-1.91)				-
Don't know	1.00 (0.52-1.91)				-
Yes	ref				-

Notes: **Model 1**: crude model; **Model 2**: adjusted for the sociodemographic factors (age, gender, geographic location, education level, income, health care card holder, car ownership); **Model 3**: plus adjusting for health-related behaviours (smokes and recreational drug status, and sexual behaviours); **Model 4**: plus adjusting for self-rated general and oral health; **Model 5** (full model): plus adjusting for self-reported HPV vaccination status. Bold were donated as statistically significant differences.

Table S7. Multivariable association between persistent of any oral HPV infection and risk factors among Indigenous Australian adults (n=473).

	Model 1	Model 2	Model 3	Model 4	Model5
	Prevalence ratio (95% CI)				
Age group (years)					
≥ 37	1.48 (0.88-2.50)	1.39 (0.82-2.36)	1.44 (0.84-2.46)	1.47 (0.84-2.58)	1.47 (0.84-2.58)
< 37	ref	ref	ref	ref	ref
Sex					
Male	0.77 (0.42-1.40)	0.81 (0.44-1.48)	0.89 (0.48-1.65)	0.92 (0.49-1.71)	0.92 (0.49-1.71)
Female	ref	ref	ref	ref	ref
Geographic location					
Non-metropolitan	**1.53 (1.00-2.63)**	1.48 (0.86-2.54)	1.58 (0.91-2.74)	1.65 (0.93-2.93)	1.65 (0.93-2.93)
Metropolitan	ref	ref	ref	ref	ref
Education level					
High school or less	1.11 (0.66-1.86)	1.08 (0.62-1.86)	1.10 (0.63-1.91)	1.02 (0.58-1.79)	1.02 (0.58-1.79)
Trade or over	ref	ref	ref	ref	ref
Income					
Centrelink	1.24 (0.69-2.23)	1.60 (0.65-3.94)	1.52 (0.64-3.62)	1.46 (0.60-3.55)	1.46 (0.60-3.55)
Job	ref	ref	ref	ref	ref
Health care card					
Yes	1.02 (0.57-1.81)	0.78 (0.33-1.86)	0.80 (0.34-1.85)	0.81 (0.34-1.92)	0.81 (0.34-1.92)
No	ref	ref	ref	ref	ref
Car ownership					
No	0.82 (0.50-1.38)	0.79 (0.45-1.37)	0.78 (0.45-1.36)	0.79 (0.44-1.40)	0.79 (0.44-1.40)
Yes	ref	ref	ref	ref	ref
Smoke status					
Current smoker	0.88 (0.52-1.51)		0.94 (0.53-1.66)	0.93 (0.52-1.67)	0.93 (0.52-1.67)
Ex-smoker	0.43 (0.15-1.23)		0.47 (0.16-1.35)	0.37 (0.11-1.22)	0.37 (0.11-1.22)
Never smoker	ref		ref	ref	ref
Recreational drug use					
Current user	1.13 (0.61-2.09)		1.14 (0.60-2.23)	1.10 (0.55-2.21)	1.10 (0.55-2.21)
Ex-user	0.63 (0.34-1.19)		0.67 (0.34-1.31)	0.63 (0.31-1.26)	0.63 (0.31-1.26)
Never use	ref		ref	ref	ref
Had oral sex					
Yes	1.10 (0.57-2.16)		-	-	-
No	ref		-	-	-
Self-rated general health					
Excel/very good/Good	0.94 (0.53-1.66)			0.95 (0.49-1.87)	0.95 (0.49-1.87)
Fair/Poor	ref			ref	ref
Self-rated oral health					
Excel/very good/Good	0.98 (0.59-1.64)			1.02 (0.57-1.82)	1.02 (0.57-1.82)
Fair/Poor	ref			ref	ref
Ever received HPV vaccination					
No	1.35 (0.51-3.58)				-
Don't know	0.88 (0.31-2.49)				-
Yes	ref				-

Notes: **Model 1**: crude model; **Model 2**: adjusted for the sociodemographic factors (age, gender, geographic location, education level, income, health care card holder, car ownership); **Model 3**: plus adjusting for health-related behaviours (smokes and recreational drug status, and sexual behaviours); **Model 4**: plus adjusting for self-rated general and oral health; **Model 5** (full model): plus adjusting for self-reported HPV vaccination status. Bold were donated as statistically significant differences.

Table S8. Multivariable association between clearance of any oral HPV infection and risk factors among Indigenous Australian adults (n=473).

	Model 1	Model 2	Model 3	Model 4	Model5
	Prevalence ratio (95% CI)				
Age group (years)					
≥ 37	0.92 (0.71-1.19)	0.95 (0.72-1.24)	0.88 (0.64-1.22)	0.92 (0.66-1.28)	0.89 (0.64-1.25)
< 37	ref	ref	ref		ref
Sex					
Male	1.06 (0.80-1.41)	1.08 (0.81-1.45)	1.00 (0.71-1.40)	1.02 (0.73-1.44)	1.00 (0.70-1.42)
Female	ref	ref	ref	ref	ref
Geographic location					
Non-metropolitan	0.87 (0.68-1.13)	0.88 (0.68-1.15)	0.81 (0.60-1.09)	0.82 (0.60-1.12)	0.82 (0.61-1.12)
Metropolitan	ref	ref	ref	ref	ref
Education level					
High school or less	0.87 (0.67-1.13)	0.85 (0.64-1.13)	0.75 (0.55-1.02)	0.76 (0.55-1.04)	0.76 (0.55-1.04)
Trade or over	ref	ref	ref	ref	ref
Income					
Centrelink	0.91 (0.69-1.21)	1.03 (0.69-1.55)	0.96 (0.61-1.51)	0.98 (0.61-1.57)	0.98 (0.61-1.57)
Job	ref	ref	ref	ref	ref
Health care card					
Yes	0.84 (0.63-1.12)	0.78 (0.53-1.15)	0.93 (0.60-1.43)	0.95 (0.61-1.49)	0.93 (0.59-1.46)
No	ref	ref	ref	ref	ref
Car ownership					
No	**1.26 (1.01-1.62)**	**1.34 (1.00-1.79)**	1.23 (0.88-1.71)	1.16 (0.83-1.65)	1.17 (0.83-1.65)
Yes	ref	ref	ref	ref	ref
Smoke status					
Current smoker	0.82 (0.61-1.12)		0.90 (0.62-1.30)	0.91 (0.62-1.32)	0.90 (0.62-1.32)
Ex-smoker	1.15 (0.79-1.69)		1.16 (0.74-1.83)	1.15 (0.72-1.85)	1.18 (0.73-1.89)
Never smoker	ref		ref	ref	ref
Recreational drug use					
Current user	1.03 (0.73-1.46)		0.96 (0.62-1.47)	0.97 (0.63-1.50)	0.98 (0.63-1.52)
Ex-user	0.93 (0.69-1.25)		0.96 (0.68-1.37)	0.98 (0.69-1.40)	0.99 (0.69-1.41)
Never use	ref		ref	ref	ref
Had oral sex					
Yes	0.95 (0.70-1.29)		0.86 (0.59-1.25)	0.88 (0.60-1.30)	0.88 (0.60-1.30)
No	ref		ref	ref	ref
Self-rated general health					
Excel/very good/Good	**1.28 (1.02-1.79)**			1.16 (0.77-1.74)	1.16 (0.78-1.73)
Fair/Poor	ref			ref	ref
Self-rated oral health					
Excel/very good/Good	1.13 (0.86-1.49)			0.99 (0.72-1.37)	0.99 (0.71-1.36)
Fair/Poor	ref			ref	ref
Ever received HPV vaccination					
No	1.13 (0.68-1.85)				1.26 (0.71-2.25)
Don't know	1.06 (0.62-1.77)				1.29 (0.72-2.33)
Yes	ref				ref

Notes: **Model 1**: crude model; **Model 2**: adjusted for the sociodemographic factors (age, gender, geographic location, education level, income, health care card holder, car ownership); **Model 3**: plus adjusting for health-related behaviours (smokes and recreational drug status, and sexual behaviours); **Model 4**: plus adjusting for self-rated general and oral health; **Model 5** (full model): plus adjusting for self-reported HPV vaccination status. Bold were donated as statistically significant differences.
